# Prognostic Factors of Returning to Work after Sick Leave due to Work-Related Common Mental Disorders: A One- and Three-Year Follow-Up Study

**DOI:** 10.1155/2015/596572

**Published:** 2015-10-18

**Authors:** Bo Netterstrøm, Nanna Hurwitz Eller, Marianne Borritz

**Affiliations:** Department of Occupational and Environmental Medicine, Bispebjerg University Hospital, Bispebjerg Bakke 23, 2400 Copenhagen NV, Denmark

## Abstract

The aim of this paper was to assess the prognostic factors of return to work (RTW) after one and three years among people on sick leave due to occupational stress. *Methods*. The study population comprised 223 completers on sick leave, who participated in a stress treatment program. Self-reported psychosocial work environment, life events during the past year, severity of the condition, occupational position, employment sector, marital status, and medication were assessed at baseline. RTW was assessed with data from a national compensation database (DREAM). *Results*. Self-reported high demands, low decision authority, low reward, low support from leaders and colleagues, bullying, high global symptom index, length of sick leave at baseline, and stressful negative life events during the year before baseline were associated with no RTW after one year. Low work ability and full-time sick leave at inclusion were predictors after three years too. Being single was associated with no RTW after three years. The type of treatment, occupational position, gender, age, and degree of depression were not associated with RTW after one or three years. *Conclusion*. The impact of the psychosocial work environment as predictor for RTW disappeared over time and only the severity of the condition was a predictor for RTW in the long run.

## 1. Introduction

Work-related common mental disorders such as stress account for a significant portion of sick leave in modern society. Stress conditions are associated with great personal suffering as well as economic problems due to sick leave [1]. Additionally, sick leave is a major risk factor for early withdrawal from the labor market [2] with reports of only 50% of people on sick leave for more than six months for mental health disorders return to work (RTW) [3]. These findings have led to growing interest in the evaluation of stress management interventions and their effect on RTW [4, 5].

A number of reviews and meta-analyses including a Cochrane review have reviewed randomized controlled trials of stress treatment programs and concluded that they are more effective at symptom reduction than no treatment. It was also determined that cognitive behavioral therapy, CBT, is particularly more effective than other therapies in reducing symptoms [6–9]. However, findings for the impact of CBT on RTW are inconsistent and do not support a significant impact of CBT on RTW [6].

The inconsistent findings for RTW as an outcome may be due to considerable heterogeneity in jurisdictional contexts such as national differences in labor market regulations and official sick leave policies, which hamper the ability to compare study findings from different countries [10, 11]. There can also be considerable heterogeneity in the individuals included in studies of RTW with regard to the course of stress development and reasons for being stressed including both private and work-related stressors and coping with stress. Many studies on the effect of stress treatment programs have included volunteers from a certain workplace or organization but have not used sick leave as inclusion criteria. This too may lead to inconclusive findings for RTW outcomes as participants may not be sufficiently impaired at inclusion to show improvement [5, 12–14].

Between 2010 and 2012, a stress treatment study was conducted at the Department of Occupational and Environmental Medicine at the Bispebjerg Hospital in Denmark. Individuals on sick leave due to stress were randomized into one of four treatment groups: (I) group-based psychodynamic therapy and body awareness; (II) individual problem solution therapy (PST) [4] with an eight-week mindfulness course; (III) control treatment of individual therapy with psychologists outside the study team (treatment as usual (TAU)); and (IV) waitlist control group (WLCG), who received PST after three months. Initially, the data were analyzed to reveal any differences in the effects of the various interventions on symptom reduction and RTW. The two interventions ((I) and (II)) led to significant improvements in symptoms compared to the waitlist group [15, 16]. In addition, the RTW rates for the intervention groups were significantly higher after three months compared to both control groups [15, 16]. We have now followed the participants for three years from inclusion to the study in order to evaluate the long-term effect of treatment and other prognostic factors measured at baseline for RTW.

## 2. Materials and Methods 

From June 2010 to December 2010, all general practitioners in the Capital Region of Denmark (1.6 million inhabitants) were invited to refer patients with work-related common mental disorder to our project. The purpose of the study and criteria for participation were described in the invitation. The inclusion criteria were as follows: the participant had to (1) be on full-time or part-time sick leave, (2) be employed or self-employed, (3) have significant symptoms of work-related common mental disorder for at least 2 months, and (4) be motivated to participate. The exclusion criteria were (1) current abuse of alcohol or psychoactive stimulants, (2) major psychiatric disorder, and (3) significant somatic disorder assumed to be the primary cause of the stress condition. Details regarding treatment and methods have been previously described [15, 16].

All procedures followed were in accordance with the Helsinki Declaration of 1975, as revised in October 2013.

### 2.1. Dependent Variable 

#### 2.1.1. RTW

Data on employment status one and three years after inclusion in the study was obtained from the DREAM database (Danish Register for Evaluation of Marginalization [10, 22]). DREAM is a registry of the Labor Market Authority of all public transfer payments. It contains data on all Danes, including those who receive economic compensation due to sick leave, unemployment, retirement, and so forth. The data on sick leave are reported as soon as the employer reports a case of sick leave of duration of two weeks or more among his or her employees. Sick leave compensation normally terminates after one year according to the regulation. Many people on sick leave thereafter are transferred to other compensation systems. Therefore, we only considered a case to have returned to the labor market if there was actually no compensation of any type at the time of census. This meant that there were two possible assessments: (1) work, that is, full-time or part-time before sick leave, or (2) case, that is, sick leave (part-time incl.), unemployment, education, maternity leave, retirement, or death. The data in the database did not allow us to distinguish between full-time and part-time sick leave. After one year, 67 pct. were at work, 17 pct. were on sick leave (full- and part-time incl.), 11 pct. were unemployed, 3 pct. were under education, and 2 pct. had retired. These figures were almost the same after three years.

### 2.2. Independent Variables

#### 2.2.1. Psychosocial Work Environment Risk Characteristics

As the work environment was believed to be the reason for sick leave, the variables describing the work environment were those believed to be of greatest significance to RTW. The explaining variables measured at baseline were the following.

We used the full scales on demands, decision authority, skill discretion, meaningfulness, predictability, reward, role clarity, justice, and social support from leaders and colleagues from the Copenhagen Psychosocial Questionnaire (COPSOQ) [17]. As part of the sessions during the intervention, the stressors were evaluated, and the participant rated the stressors in collaboration with the therapist. Ratings ranged from 1 = no or low influence to 4 = very high influence. The possible work-related stressors were bad management, bad work environment, reorganization, and work pressure. The scores from the work-related stressors were summed and divided by four to calculate the work environment factor index (maximum score 4). The participants were also asked whether bullying was a stressor. This issue was not included in the averaged measure because we find this stressor very much different from reorganization and work pressure.

#### 2.2.2. Life Events during the Last Year

However, it is well known that people suffering from stress experience stressors in both their work and their private lives [18, 19]. The baseline questionnaire also included information about life events during the last year, that is, problems with colleagues, getting fired, death in family, divorce, and economic troubles (yes/no). Life events were summed and divided by five to calculate the life event one-year score (maximum score 5).

#### 2.2.3. Demographics

Gender, age, occupational position (blue, white collar, or academic worker), employment in the public or private sector, and marital status were recorded.

The type of treatment in the program was also used as an independent variable.

#### 2.2.4. Seriousness of Stress Conditions

The seriousness of stress conditions was estimated in several ways.

- The form of sick leave, that is, being on full-time sick leave at time of inclusion or not and number of days on sick leave before attending the project.
- Work ability, measured on a scale ranging from 0 to 10 using the following question: “Assess your work ability as ten points when you are at your best. How do you rate your work ability currently?”
- The degree of stress, measured using the following question: “‘Stress' is a condition characterized by unrest, agitation, or anxiety and/or sleeping problems. Do you experience stress at the moment?” There were five options for answers ranging from “not at all” to “always” [20].
- The SCL92, a validated scale on 92 questions on psychological symptoms, used to calculate the global symptom index (GSI) and its nine subscales according to [21].
- Medication with antidepressants at baseline (yes/no).
- Alcohol consumption.

### 2.3. Statistical Analyses

First, an analysis of the differences between the participants of the study, the excluded and the drop-outs, was conducted as well as the mean and standard deviation (SD) of the demographic variables to describe the participants. Next two sets of analysis were conducted. First, the participants at work (*N* = 150) were compared to cases (*N* = 73) after one year. Next, those at work at both the one- and the three-year census (*N* = 111) were compared to those who were cases at both times (*N* = 32). A Student's *t*-test was used to evaluate the differences in continuous variables, and a chi-square test was used in connection with the categorical variables. A series of bivariate logistic regression analyses were conducted to reveal if the variables were significantly associated with the outcome. Correlations of the explanatory variables, covariates, and outcome were analyzed to reveal any multicollinearity between the variables. Next, several multivariate logistic regression analyses with RTW at one- and three-year follow-up as dependent variable were conducted. The included independent variables were chosen so that multicollinearity was not present. The chosen variables were decision authority, bullying, work ability index, and full-time/part-time sick leave at baseline. In model 1, the adjustment factors were age, gender, marital status, and occupational position. Model 2 included GSI and model 3 in addition life events. These analyses were repeated in general linear models (GLM) in order to evaluate any interactions between the independent variables. Finally, in model 4 multivariate logistic regression analyses were conducted with the four chosen independent variables forced into the model at the same time.

## 3. Results

From August 4, 2010, to April 8, 2011, 320 potential participants were referred to the study, of which 268 fulfilled the inclusion criteria. The procedure of randomization of treatment has been previously described [15]. However, only 223 individuals completed the treatment (87.8%). Twelve people did not show up or decided that they did not want to participate (drop-outs). Of the remaining 33, 17 were excluded due to major psychiatric disorder during the first weeks of treatment, and 16 participants were excluded because they did not complete the treatment or were absent more than two times during the duration of treatment.  Table 1 shows the characteristics of the participants in the intervention group compared to the individuals who were excluded or did drop out. The excluded persons were predominately women, were less educated, and had higher symptom level scores but were on the other variables comparable to the intervention group.

At one-year follow-up, 150 participants were working full-time, whereas 73 were not. After three years, 111 of the 150 participants were still working, whereas only 32 were being a case both years. The results of the one-year follow-up of the bivariate *t*-tests including one explanatory variable at a time with RTW as a dependent variable showed that several work environment risk factors were significantly associated with not being at work after one year. High demands, low decision authority, low reward, low support from leaders and colleagues, and being bullied were all self-reported baseline risk factors among those not being at work. The work environment index and life events one-year index were associated with no RTW. The seriousness of the condition in the form of GSI, work ability index, full-time sick leave at inclusion, and number of days on sick leave before inclusion was significantly greater in the group that had not returned at work after one year (Table 2).

In contrast, the only factors significantly associated with being a case after both one and three years were being on full-time sick leave at baseline, low work ability index score, and being single.

The correlation analysis of data shown in Table 3 demonstrated that the number of days of sick leave at time of inclusion in the study was not correlated with any of the scales measuring degree of symptoms. The scales measuring degree of symptoms were all significantly intercorrelated. In addition, the different measures of work environment were correlated, and the data on symptom degree were correlated with scales of work environment. Therefore, the data set showed multiple collinearity problems.

The multivariate logistic regression analyses showed that high work ability index, bullying, and full-time sick leave prior to inclusion in the study were significantly associated with RTW after one year after full adjustment (Table 4). Low decision authority remained significant after adjustment for age, gender, and occupational position, but not after further adjustments. No interactions between the independent variables were found in the GLM-analyses. However, only work ability remained significant with RTW after one year, when the chosen independent variables were forced into the model with full adjustments (Table 4). Demands and social support from leaders as well as colleagues were significantly associated with RTW after adjustment for age, gender, and occupational position whereas the predictability and rewards were only borderline significant (0.1 > *P* > 0.05) (data not shown). Life events were not significantly associated with RTW after adjustment for age, gender, and occupational position.

After three years only full-time sick leave at baseline and low work ability were significantly negatively associated with RTW even after adjustments as above (data not shown).

## 4. Discussion

In this study of predictors of being at work one year after inclusion in a stress treatment project, we determined that self-reported psychosocial work environment, life events, part-time sick leave, and length of sick leave at baseline were of significance to being at work, whereas type of treatment, employment grade, degree of depression, and life events were not. The association between psychosocial work environment factors and RTW disappeared after three years.

The outcome was “being at work or not.” The data to achieve the outcome were collected from DREAM, a national register of public compensation. However, the validity of these data may be questioned [10, 11, 22]. The participants who were in the “not being at work” group may be very different as some are in fact still disabled from stress, whereas others are on education, have retired, or are not employed. It was not possible to select only those still disabled due to stress due to the categories in the register. The results should therefore be read as predictors for “being at work or being something else” and not “being at work or being disabled by stress.” A bad work environment may indeed be a reason for wanting to pursue further education or retire, but nonetheless a positive working environment predicts RTW after one year.

The most important result was that working environment has significance to an early RTW. Those who were not returned to the workplace one year after inclusion in the project had reported significantly more demands and less decision authority, reward, and support at baseline, than the group which was working full-time after one year. The chance of getting back is larger if you felt comfortable at work before getting stressed. An early return to work is important as this prevents withdrawal from the labor market [2, 3]. Also, a successful RTW-process is a success to the workplace and may prevent other cases of long time sick leave. When the process has a positive result this will spread in the organization. However, when the working environment is bad the chance of getting back is smaller, the RTW-process is hard, and you may be squeezed out of the workplace. This could not be demonstrated by the analyses including only the participants who were full-time workers at both one and three years compared to those who were cases both years. The data from the DREAM database did not give us the opportunity to analyze part-time employees separately. However, those who were cases at both years had a nonsignificant tendency of experiencing high demands at baseline.

We chose to include variables related to life events, as conditions in private life may also be of significance to RTW and may delay RTW. However, it was not possible to reveal any significance of these variables as they relate to RTW.

The self-reported psychosocial working environment was of some significance to the outcome, as high demands, low decision authority, bullying, low rewards, and low social support from leaders and colleagues were all significantly associated with “not being at work.” However, after adjustment for severity of the condition, these associations were insignificant. The seriousness of the stress condition in the form of number of days on sick leave, being on full-time sick leave, GSI, and most pronounced work ability index was also significantly associated with RTW. A recent Danish paper has reported similar results on the association between depressive symptoms and long-term sickness absence, but in that study poor psychosocial work environment did not predict sickness absence [23]. However, the psychosocial work environment was assessed by the use of unit level aggregated measures on work environment. Though structural conditions may be the same for several individuals working in the same unit of an organization, the work environment may be perceived very differently by single individuals. This difference in perception may be the reason that our findings differ from the findings by Hjarsbech et al.

Low social support from leaders, low social support from colleagues, and bullying were all associated with RTW after one year. In line with this finding are the findings by Arends et al. that associate conflicts with a superior with recurrent sick leave [4]. If you expect problems at the workplace, it is of course not easy to return.

The finding that the type of treatment was not associated with RTW after one and three years is in accordance with earlier findings [6]. This might reflect the fact that although treatment accelerates the RTW-process, the severity of the condition and other factors are more important in the long run [4, 5, 11].

In this study, the severity of the disorder (full-time sick leave and poor self-rated work ability) was found to be the main predictor in the long run in addition to being single, which is a main finding in many studies on the relation between marital status and disease.

Our results may be questioned, as the study has several weaknesses. The study size is rather small, including only 223 participants who fully completed the study. This includes the risk of determining findings by chance, as a small number of participants may completely change the results. However, the strength of the study is the well-validated outcome and extensive exposure measures.

## Figures and Tables

**Table 1:** Analyses of excluded participants and drop-outs compared to those who fulfilled treatment. Excluded comprises those excluded due to major psychiatric disease (*N* = 17) and absence from treatment (*N* = 16). Drop-outs are those who did not attend the treatment at all even though they fulfilled the inclusion criteria.

	Intervention	Excluded	*P*	Drop-outs	*P*
*N*	223	33		12	
Age, mean (SD)	44.2 (8.8)	43.2 (10.8)	0.60	40.9 (7.7)	0.19
Women %	80.3	65.2	0.08	66.7	0.21
Blue collar workers %	34.3	30.8	0.95	(NA)	(NA)
Married %	62.2	53.8	0.37	(NA)	(NA)
Global symptom index (GSI) (SCL92), mean (SD)	1.23 (0.54)	1.66 (0.75)	0.005	(NA)	(NA)
Depression score (SCL92), mean	1.74 (0.76)	2.42 (1.02)	0.002	(NA)	(NA)
At work after 1 year %	67.3	51.8	0.20	41.7	0.07
At work after 3 years %	68.9	54.5	0.31	34.8	0.04

**Table 2:** Bivariate associations between independent factors at baseline and RTW full-time or not one and three years after baseline.

	One year			Both one and three years		
	Full-time job	Case	*P*	Full-time job	Case	*P*
*N*	150	73		111	32	
Demographics						
Age, mean (SD)	44.8 (8.6)	42.8 (9.7)	0.14	44.7 (7.8)	43.6 (11.1)	0.52
Women %	79.3	82.2	0.38	74.6	70.7	0.68
Blue collar/white collar/academic worker %	34.9/49.7/15.6	37.3/53.3/9.3	0.41	30.6/54.8/14.5	39.5/52.6/7.9	0.42
Public sector %	68.4	71.2	0.71	68.2	74.5	0.46
Married %	64.6	57.1	0.30	62.4	35.9	0.005
Alcohol (U/week), mean (SD)	4.3 (5.1)	3.6 (4.6)	0.29	4.3 (4.5)	3.9 (5.0)	0.66
Treatment group %						
I	70.0	30.0	0.87	80.4	19.6	0.68
II	63.8	36.2		71.2	28.6	
TAU (III)	64.8	35.2		71.8	28.2	
WLCG (IV)	65.5	34.5		70.6	29.4	
Psychosocial work environment						
High demands, mean (SD)	61.9 (12.4)	66.6 (15.5)	0.02	61.4 (12.2)	65.6 (15.6)	0.11
Low decision and authority, mean (SD)	56.1 (21.7)	64.7 (22.0)	0.007	56.6 (21.5)	62.8 (22.2)	0.16
Low skill discretion, mean (SD)	32.5 (16.0)	35.3 (16.5)	0.24	32.3 (15.5)	35.9 (16.2)	0.24
Low meaningfulness, mean (SD)	32.1 (19.6)	32.0 (21.2)	0.98	32.2 (20.5)	32.2 (20.9)	0.99
Low predictability, mean (SD)	55.1 (23.5)	61.6 (22.9)	0.06	56.6 (23.0)	56.6 (21.1)	0.91
Low reward, mean (SD)	49.7 (24.2)	57.4 (24.7)	0.03	51.9 (24.1)	57.2 (26.9)	0.29
Low role clarity, mean (SD)	43.5 (22.3)	42.0 (22.4)	0.67	44.1 (22.3)	37.2 (18.2)	0.11
Low support from leader, mean (SD)	52.3 (24.9)	60.2 (26.4)	0.03	53.6 (25.0)	56.4 (27.7)	0.57
Low degree of justice, mean (SD)	55.3 (20.4)	59.0 (19.8)	0.21	57.3 (20.8)	60.2 (20.1)	0.50
Bullying, mean (SD)	1.4 (0.9)	1.7 (1.1)	0.05	1.4 (0.9)	1.6 (1.1)	0.41
Work environment index (4 items), mean (SD)	2.3 (0.4)	2.4 (0.6)	0.05	2.2 (0.4)	2.3 (0.5)	0.50
Life events last year score (5 items), mean (SD)	1.3 (1.9)	1.4 (2.3)	0.02	1.5 (1.8)	1.5 (1.8)	0.89
Severity of condition						
Global symptom index (GSI) (SCL92), mean (SD)	1.2 (0.5)	1.3 (0.5)	0.05	1.23 (0.5)	1.3 (0.6)	0.35
Somatic symptom score (SCL92), mean (SD)	1.3 (0.8)	1.5 (0.8)	0.17	1.4 (0.8)	1.5	0.15
Depression score (SCL92), mean (SD)	1.7 (0.8)	1.8 (0.8)	0.56	1.7 (0.8)	1.8 (0.8)	0.57
Anxiety score (SCL92), mean (SD)	1.3 (0.7)	1.4 (0.7)	0.19	1.4 (0.7)	1.4 (0.7)	0.98
Antidepressant medication %	15.9	18.8	0.69	15.2	17.2	0.78
Work ability index, mean (SD)	2.8 (2.2)	1.8 (2.0)	0.001	2.9 (2.2)	1.7 (2.1)	0.04
Stress, mean (SD)	3.7 (0.9)	4.0 (0.9)	0.11	3.8 (0.9)	4.1 (0.9)	0.19
Full-time sick leave at baseline %	62.5	76.8	0.03	61.5	82.1	0.02
Days with full-time sick leave at baseline, mean (SD)	40.9 (49.4)	57.0 (80.9)	0.04	43.6 (51.2)	46.4 (58.4)	0.68

TAU: treatment as usual (III).

WLCG: wait list control group (IV).

**Table 3:** Correlations between RTW after one year and independent variables at baseline.

	1	2	3	4	5	6	7	8	9	10	11	12	13	14
1 RTW after one year	1	**−0.160**	***−0.181***	−0.130	**−0.146**	**−0.147**	**−0.141**	**−0.171**	−0.124	−0.133	***0.208***	−0.103	**−0.145**	**−0.123**
2 High demands		**1**	***0.194***	**0.134**	**0.156**	**0.150**	0.078	**0.186**	−0.066	−0.028	−0.083	***0.210***	0.054	0.045
3 Low decision authority			1	***0.351***	***0.381***	***0.253***	***0.232***	***0.296***	−0.006	***0.259***	−0.032	***0.194***	0.053	−0.084
4 Low predictability				1	***0.603***	***0.609***	***0.397***	**0.211**	0.117	**0.187**	−0.086	***0.222***	***0.176***	**0.147**
5 Low rewards					1	***0.737***	***0.486***	***0.265***	***0.246***	***0.276***	−0.044	***0.217***	***0.182***	0.067
6 Low support from leader						1	0.447	0.189	0.130	0.195	−0.101	0.165	**0.160**	0.094
7 Low support from colleagues							1	**0.182**	***0.215***	***0.232***	−0.063	0.110	−0.130	0.096
8 Work environment index								1	***0.205***	***0.389***	−0.137	0.248	0.001	−0.015
9 Life event index									1	***0.202***	−0.008	**0.197**	0.045	−0.028
10 Bullying										1	0.031	**0.179**	−0.069	−0.130
11 High work ability index											1	***−0.253***	***−0.304***	−0.079
12 Global symptom index												1	0.079	−0.075
13 Full-time sick leave at baseline													1	**0.182**
14 Days of sick leave at baseline														1

Bold: *P* < 0.05. Bold and italic: *P* < 0.01.

**Table 4:** Logistic regression analyses of prognostic variables for RTW after one year.

Prognostic variables	Model 1	Model 2	Model 3	Model 4
	OR (95% CI)	OR (95% CI)	OR (95% CI)	OR (95%)
Low decision authority	0.982 (0.970–0.999)	0.987 (0.971–1.004)	0.986 (0.971–1.002)	0.990 (0.975–1.007)
Bullying	0.715 (0.513–0.994)	0.677 (0.474–0.966)	0.634 (0.426–0.926)	0.731 (0.513–1.040)
Work ability index	1.220 (1.038–1.434)	1.285 (1.074–1.537)	1.356 (1.113–1.612)	1.235 (1.029–1.401)
Full-time sick leave at baseline	0.431 (0.222–0.830)	0.430 (0.223–0.830)	0.454 (0.234–0.880)	0.511 (0.251–1.010)

In models 1–3, the prognostic variable was analyzed separately with adjustment for the following.

Model 1: age, gender, marital status, and occupational position.

Model 2: model 1 and global symptom index.

Model 3: model 2 and life events.

Model 4: all four prognostic variables forced into the model adjusted for age, gender, occupational position, global symptom index, marital status, and life event last year.
