# Corrigendum to “Prognostic Factors of Returning to Work after Sick Leave due to Work-Related Common Mental Disorders: A One- and Three-Year Follow-Up Study”

**DOI:** 10.1155/2016/8305414

**Published:** 2016-07-25

**Authors:** Bo Netterstrøm, Nanna Hurwitz Eller, Marianne Borritz

**Affiliations:** Department of Occupational and Environmental Medicine, Bispebjerg University Hospital, Bispebjerg Bakke 23, 2400 Copenhagen NV, Denmark

In the Materials and Methods section in the article titled “Prognostic factors of returning to work after sick leave due to work-related common mental disorders: A one- and three- year follow-up study,” [1] the sentence “All procedures followed were in accordance with the Helsinki Declaration of 1975, as revised in October 2013” should be corrected to “The randomization procedure (i.e. drawing lots) was conducted by a secretary who was located at another hospital. The outcome of the draw was stated in the referral document and sent to the Bispebjerg Hospital where the assessment interview took place. Interviews of participants randomized to the WLCG were conducted by a trained medical physician or a psychologist, who was able to address any disappointments of the participants and thereby attempt to prevent biases in the responses to the questionnaires. Assessment interviews involving the other participants were conducted by psychology students. The participants were informed of their group assignment after they had agreed to enter the project and they had signed the written informed consent.”

## References

[1] Netterstrøm B., Eller N. H., Borritz M. (2015). Prognostic factors of returning to work after sick leave due to work-related common mental disorders: a one- and three-year follow-up study. *BioMed Research International*.

